# Targeted Identification of Rice Grain-Associated Gene Allelic Variation Through Mutation Induction, Targeted Sequencing, and Whole Genome Sequencing Combined with a Mixed-Samples Strategy

**DOI:** 10.1186/s12284-022-00603-2

**Published:** 2022-11-03

**Authors:** Kai Sun, Dandan Li, Aoyun Xia, Hua Zhao, Qin Wen, Sisi Jia, Jiafeng Wang, Guili Yang, Danhua Zhou, Cuihong Huang, Hui Wang, Zhiqiang Chen, Tao Guo

**Affiliations:** National Engineering Research Center of Plant Space Breeding, South China Agricultural University, 510642 Guangzhou, People’s Republic of China

**Keywords:** Rice grain type, Heavy-ion irradiation, Allelic variation, Targeted sequencing, Whole genome sequencing

## Abstract

**Background:**

The mining of new allelic variation and the induction of new genetic variability are the basis for improving breeding efficiency.

**Results:**

In this study, in total, 3872 heavy ion-irradiated M_2_ generation rice seeds and individual leaves were collected. The grain length was between 8 and 10.22 mm. The grain width was between 1.54 and 2.87 mm. The results showed that there was extensive variation in granulotype. The allelic variation in *GS3* and *GW5* was detected in 484 mixed samples (8:1) using targeted sequencing technology, and 12 mixed samples containing potential mutations and 15 SNPs were obtained; combined with Sanger sequencing and phenotype data, 13 key mutants and their corresponding SNPs were obtained; protein structural and functional analysis of key mutants screened out 6 allelic variants leading to altered grain shape, as well as the corresponding mutants, including long-grain mutants *GS3-2* and *GS3-7*, short-grain mutants *GS3-3* and *GS3-5*, wide-grain mutant *GW5-1* and narrow-grain mutant *GW5-4*; whole genome sequencing identified new grain length gene allelic variants GS3-G1, GS3-G2 and GS3-G3.

**Conclusion:**

Based on the above studies, we found 6 granulotype mutants and 9 granulotype-related allelic variants, which provided new functional gene loci and a material basis for molecular breeding and genotype mutation and phenotype analysis. We propose a method for targeted identification of allelic variation in rice grain type genes by combining targeted sequencing of mixed samples and whole genome sequencing. The method has the characteristics of low detection cost, short detection period, and flexible detection of traits and genes.

**Supplementary Information:**

The online version contains supplementary material available at 10.1186/s12284-022-00603-2.

## Background

Rice (*Oryza sativa* L.) is one of the three major food crops and is a staple food for nearly half of the world’s population (Miura et al. 2010). Excellent germplasm resources are the basis for improving the breeding efficiency of new rice varieties (Zhao et al. 2011). Various physical or chemical mutagenic factors can induce changes in biological genetic material, resulting in new allelic variations and species. As a new radiation mutagenesis method, heavy ion mutagenesis has unique advantages, such as a high mutation rate, wide mutation spectrum, fast mutation stability, and stable and reliable mutagenesis, and this method is simple and easy to implement (Qu et al. 2007; Hase et al. 2012). Due to the high linear energy transfer (LET) properties of heavy ion beams, single nucleotide variations (SNVs) and insertions/deletions (InDels) and structural variations (SVs) can be induced at higher frequencies (Zheng et al. 2021). This method can induce heritable variation in plant genomes in contemporary times. The resulting mutants are important materials for functional genomics research (Oono et al. 2020). Heavy ion radiation is one of the effective ways to innovate rice germplasm (Jing et al. 2021). In recent years, this technique has played an important role in plant breeding (Yang et al. 2019a; Li et al. 2019; Sjahril et al. 2020; Okasa et al. 2021; Zhang et al. 2022).

Mining of allelic variations is the key to creating new germplasm for plants and animals. The rice *Wx* gene is the main gene that controls amylose synthesis, and the discovery and utilization of its allelic variation is an important way to analyze rice quality variation and is also an important basis for rice quality improvement. Currently, the *Wx* gene has been discovered and identified, and multiple important allelic variants, including *Wx*^*lv*^, *Wx*^*a*^, *Wx*^*b*^, *Wx*^*in*^, *Wx*^*mp*^, *Wx*^*op*^, and *wx* (Zhang et al. 2019), have also been identified. *GS3* is a major QTL controlling grain length in rice, and the protein it encodes negatively regulates grain length (Fan et al. 2006; Mao et al. 2010) identified the four alleles *GS3*:*GS3-1*, *GS3-2*, *GS3-3* and *GS3-4*, in which *GS3-3* has an SNP mutation in exon 2, resulting in a long grain; *GS3-4* has a 1-bp deletion at 357 bp, resulting in a decrease in long grain length. *GW5* is a major QTL that controls grain width in rice. The 1212 bp deletion in wide-grain varieties regulates the expression of *GW5* and then the size of the grain. Using CRISPR technology to delete approximately 5 kb downstream of *GW5* can increase the grain width and size. Moreover, grain weight can increase yield (Liu et al. 2017).

For the screening and identification of allelic variants, in classical forward genetics studies, complex mapping populations and linkage maps are usually constructed, and genetic markers are used for gene linkage analysis (Serquen et al. 1997; Zhang et al. 2012; Fazio et al. 2003). The targeted induced local lesions in genomes (TILLING) technique is a reverse genetics technique developed in the 1990s. This technique is based on chemical mutagenesis materials and combines chemical mutagenesis technology with PCR screening technology and high-throughput detection methods. Linked together, a high-throughput and rapid detection of point mutations in target gene regions has formed a technical system (Henikoff and Comai 2003) that has been applied in a variety of plants and promoted mutagenesis and breeding development (Boualem et al. 2014; Anai 2012; Ochiai et al. 2011; Chen and Dubcovsky 2012; Minoia et al. 2010). MutMap is a forward genetic gene mapping strategy and genetic analysis method developed based on whole genome sequencing (WGS) (Abe et al. 2012). The MutMap method also includes a variety of developments and extensions, such as MutMap+ and MutMap-Gap. These methods do not require the establishment of cumbersome progeny mapping groups and do not rely on genetic hybridization and any linkage information. The identification process of variant loci has been successfully applied to the study of multiple gene mappings in different species (Takagi et al. 2013a, b, 2015; Rym et al. 2017). In recent years, targeted sequencing technology has been widely used. Targeted sequencing is GenoPlexs based on multiplex PCR and GenoBaits based on liquid-phase probe capture, which can detect multiple SNPs within a single amplicon, greatly improving intratarget variation and detection efficiency. This technique has the characteristics of high marker flexibility and high detection efficiency and can be widely used in biological evolution, genetic map construction, gene location cloning, marker trait association detection, allelic variation detection, etc. (Shen et al. 2021; Guo et al. 2019; Lu et al. 2019; Li et al. 2020; Du et al. 2019; Yang et al. 2019b).

**Fig. 1:**
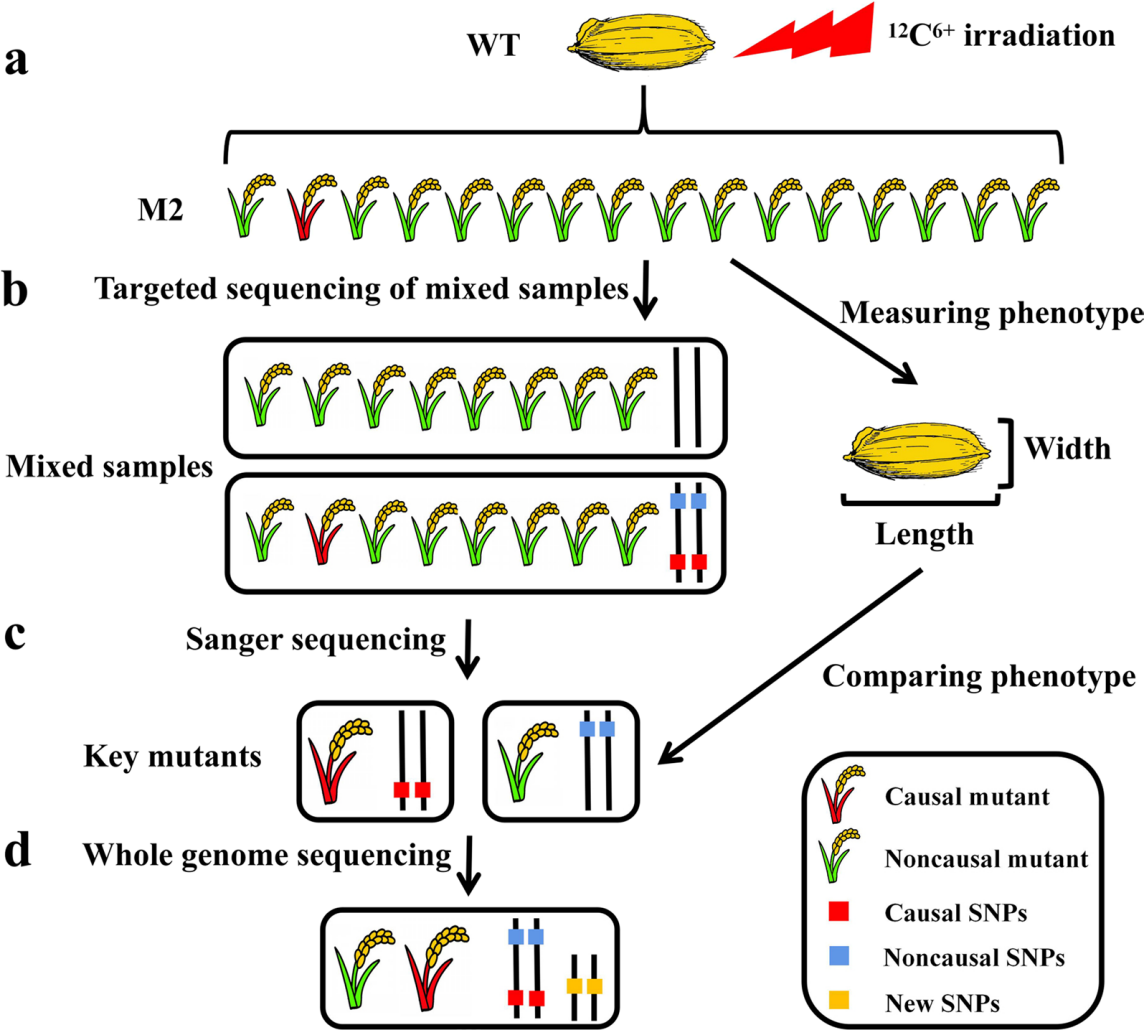
Simplified steps of the MTWA process. **a** Wild-type (WT) seeds were irradiated with a carbon ion beam, and the M_2_ generation was planted and harvested. **b** The M_2_ generation mixed samples were constructed at a ratio of 8:1 for targeted sequencing detection, and the grain shape of the M_2_ generation seeds was measured. **c** Sanger sequencing was performed on individual plants in a mixed sample of potential mutations detected by targeted sequencing and compared with phenotypes to identify key mutants. **d** WGS of key mutants to resolve associations between mutant phenotypes and genotypes and to mine for novel allelic variants

In this study, 3872 ^12^C^6+^ radiation mutagenized mutant materials for the second generation were identified by mixed sample targeted sequencing technology, and the mutant mixed samples and SNPs related to the granulotype genes *GS3* and *GW5* were mined and then selected by Sanger sequencing. Mutant individual plants, combined with phenotype and protein function analysis, were utilized to further select key mutant individual plants and conduct WGS to analyze the relationship between mutant phenotype and genotype, discover new allelic variations, and establish a system. An efficient and accurate method for directional identification of allelic variation in rice grain type genes through mutation induction, targeted sequencing, and whole genome sequencing combined with a mixed-samples strategy, abbreviated as MTWA, was developed (Fig. 1). The innovation of this method is that the use of targeted sequencing combined with WGS can quickly screen mutants and identify mutation sites, with high detection throughput, low detection cost, and flexible target traits and sites.

## Results

### Grain Typing Phenotype Investigation

**Fig. 2:**
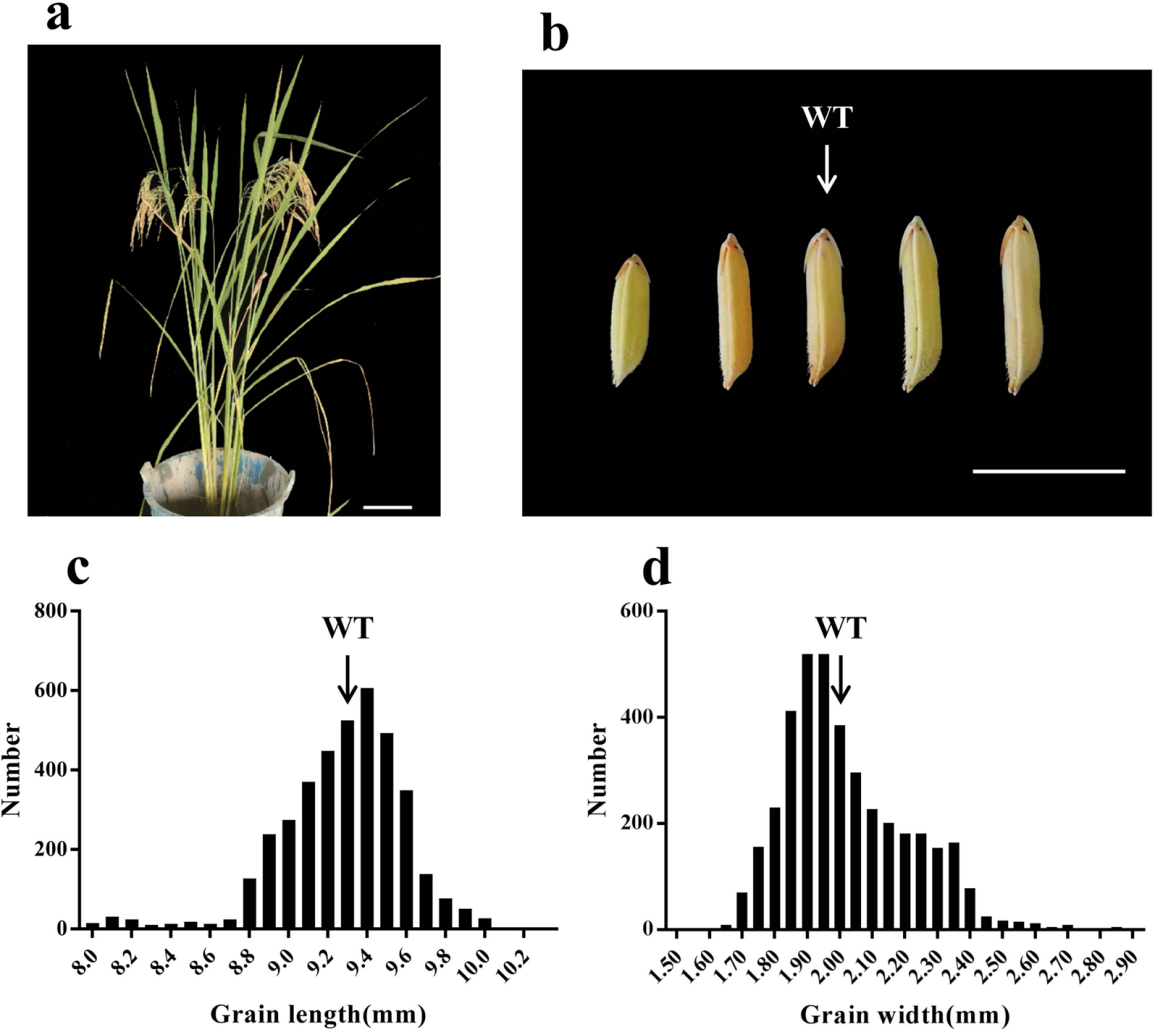
**a** WT Huahang 31 plant type. Scale bars = 10 cm. **b** Mutant granulotype and WT alignment. Scale bars = 1 cm. **c** Material grain length number distribution. **d** Material grain width number distribution

The wild-type (WT) material used in this study was Huahang No. 31 (Fig. 2a). A total of 3872 seeds of the M_2_ generation per plant were harvested. We measured the grain length and width of these seeds (Additional file 7; Table S4). The grain length was between 8 and 10.22 mm, the average length was 9.28 mm, and the coefficient of variation was 1.89%. The grain length of WT was 9.31 mm (Fig. 2c). The grain width was between 1.54 and 2.87 mm, with an average width of 2.02 mm, and the WT grain width was 2.03 mm, with a coefficient of variation of 0.56% (Fig. 2d, Table 1). Both the grain length and grain width conformed to a normal distribution and had a wide variation range. Compared with the WT, there were many materials with large grain type differences, indicating that there were several potential grain type mutations (Fig. 2b).

**Table 1:** Grain type phenotype survey

trait	Min	Max	Average	Standard deviation	Coefficient of variation/%
Length (mm)	8.00	10.22	9.28	0.18	1.89
Width (mm)	1.54	2.87	2.02	0.01	0.56

### Targeted Sequencing to Screen a Mixed Pool of Potential Mutations

**Fig. 3:**
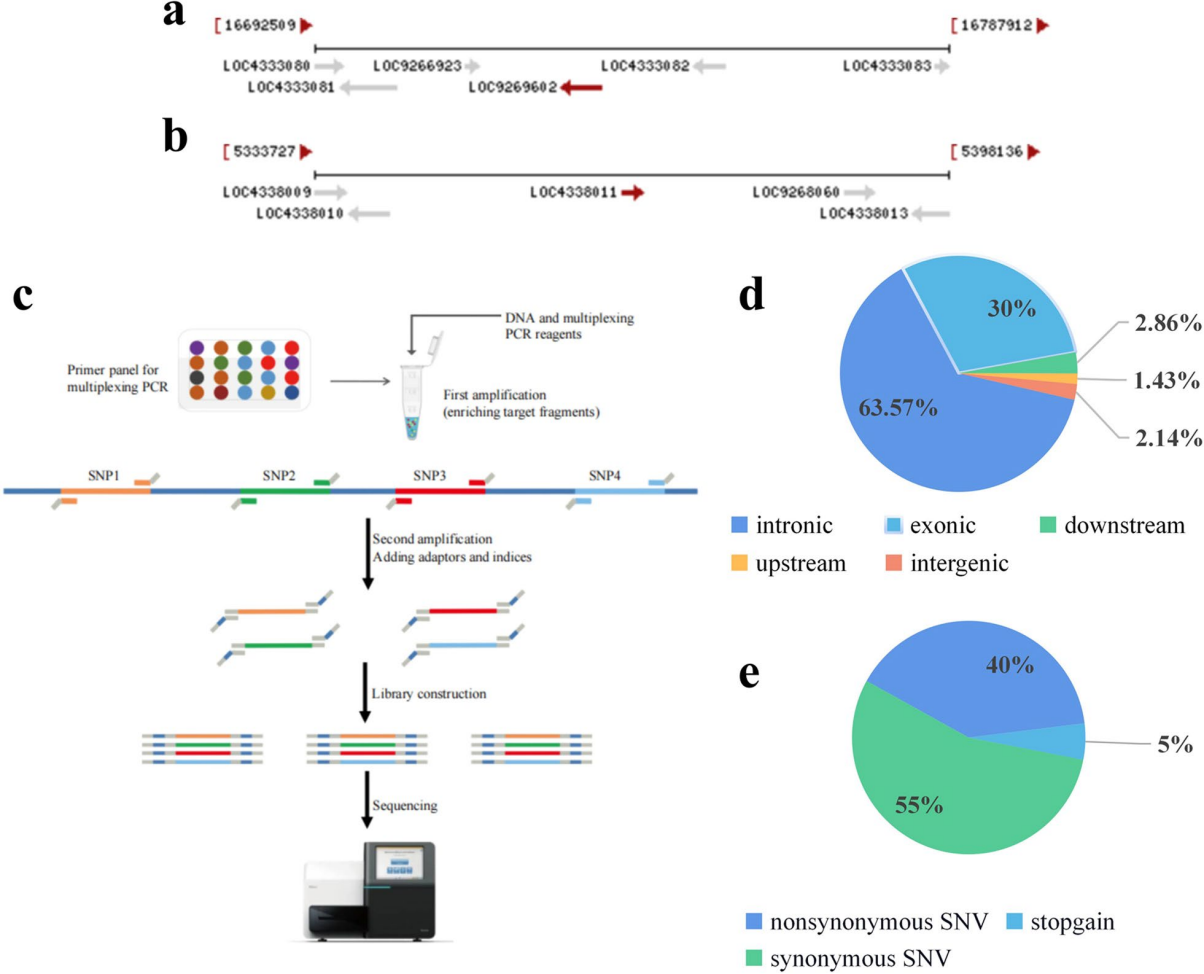
**a** *GS3* gene information. **b** *GW5* genetic information. **c** Targeted sequencing process. **d** Location distribution of SNPs in the genome. **e** Mutation site functional type distribution

The target genes of targeted sequencing are *GS3* (Fig. 3a) and *GW5* (Fig. 3b). *GS3* and *GW5* are the two genes that have the greatest influence on grain length and grain width, and their mechanism has been thoroughly studied. Targeted sequencing (Fig. 3c) detected a total of 179 mutation sites were obtained (Additional file 8; Table S5), all of which were homozygous mutations, of which 110 sites were in the *GS3* interval and 69 were in the *GW5* interval. The total mutation frequency in the *GS3* interval was calculated to be 4.05 × 10^− 5^, and the total mutation frequency in the *GW5* interval was 9.02 × 10^− 5^ (total mutation frequency = mutation base number/gene fragment length). Among the 179 mutation sites, 63.57% were located in the intron region, and 30% were located in the exon region (Fig. 3d, Additional file 9; Table S6). We retained only the nonsynonymous and nonsense mutations located in the exon region that could cause amino acid changes. At the same time, reliable sites with relatively high reads were screened, and a total of 15 SNPs were obtained (Table 2), of which 14 were nonsynonymous mutations and 1 was a nonsense mutation, including 11 *GS3* interval loci and 4 *GW5* interval locus points (Fig. 3e), for a total of 12 mixed samples. Of the 12 mixed samples, 8 were associated with *GS3* and 4 were associated with *GW5*. 4–101, 6–44 and 7–78 each contained two SNPs, and the remaining nine mixed samples only had one SNP.

**Table 2:** Targeted sequencing mutation site information

SNP number	Mixed sample number	Gene	Location	Ref	Mut	Function type
SNP-1	4–101	*GS3*	16,732,992	G	C	nonsynonymous SNV
SNP-2	4–101	*GS3*	16,734,441	G	T	stopgain
SNP-3	5–6	*GS3*	16,734,009	G	C	nonsynonymous SNV
SNP-4	6–23	*GS3*	16,731,920	C	A	nonsynonymous SNV
SNP-5	6–44	*GS3*	16,729,753	A	T	nonsynonymous SNV
SNP-6	6–44	*GS3*	16,729,861	G	T	nonsynonymous SNV
SNP-7	7–112	*GS3*	16,735,064	G	C	nonsynonymous SNV
SNP-8	7–41	*GS3*	16,729,815	C	T	nonsynonymous SNV
SNP-9	7–78	*GS3*	16,729,903	G	A	nonsynonymous SNV
SNP-10	7–78	*GS3*	16,729,863	C	A	nonsynonymous SNV
SNP-11	9−5	*GS3*	16,729,886	C	A	nonsynonymous SNV
SNP-12	5–35	*GW5*	5,365,411	A	G	nonsynonymous SNV
SNP-13	7–82	*GW5*	5,366,479	C	G	nonsynonymous SNV
SNP-14	10–14	*GW5*	5,366,520	T	G	nonsynonymous SNV
SNP-15	10–51	*GW5*	5,366,501	A	G	nonsynonymous SNV

### Screening of Individual Mutant Plants and Identification of Their Authenticity

**Fig. 4:**
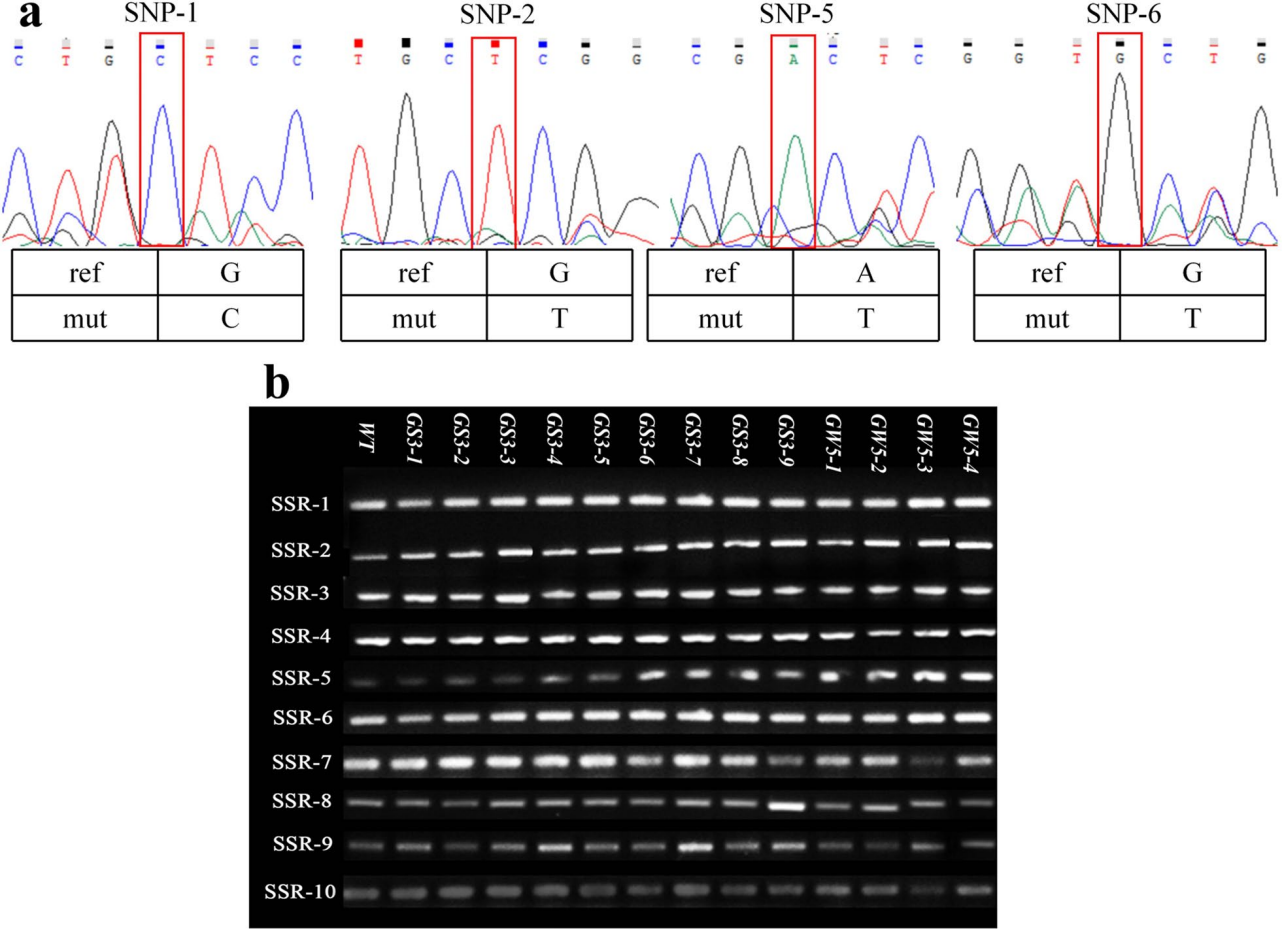
**a** Sanger sequencing results; the red box is the target SNPs. The Sanger sequencing results of SNP-1 and SNP-2 were consistent with the targeted sequencing results. SNP-5 and SNP-6 were consistent with WT, and no variants were detected. **b** Results of agarose gel electrophoresis. The 10 SSR fragment polymorphisms of 13 mutant individual plants were consistent with *WT*

To further screen out the mutant individual plants from the mixed samples, we isolated individual plants in the 12 mixed samples, which contained a total of 96 individual plant materials. The 96 individual plant materials of the fragment were subjected to Sanger sequencing and compared with the results of targeted sequencing to determine the target mutant individual plant. A total of 13 loci were consistent with the targeted sequencing results, among which the Sanger sequencing results of SNP-5 and SNP-6 were different from the targeted sequencing results (Fig. 4a), so the mutants at these two loci were excluded and targeted sequencing. The concordance rate with Sanger sequencing was 86.67%, and a total of 13 mutants were screened. The complete 15 SNP results are shown in Additional file 2: Fig. S2.

To verify the authenticity of the selected mutants, we identified the authenticity of the selected 13 single-plant materials according to the technical regulations for the identification of rice varieties (SSR marking method) issued by the Ministry of Agriculture (NY/T 1433–2014) and designed a total of 10 pairs of SSR markers (Additional file 6: Table S3). The agarose gel electrophoresis detection results of the 13 mutant individual plants were consistent with the *WT*, indicating that they were all true mutations (Fig. 4b).

### Phenotypic Verification and Protein Function Analysis of Individual Mutant Plants

After verification, the corresponding grain type and phenotype data of the real variant individual plants were found according to the number. Only 6 of the 13 individual plants showed significant changes in grain type, and the grain types of the remaining 7 individual plants were the same as those of the control. There was no significant difference in the ratios between samples (Table 3). According to the screening results of targeted sequencing, 13 SNPs are nonsynonymous and nonsense mutations, which theoretically lead to amino acid changes, while some SNPs do not cause significant changes in phenotype, presumably not changing the function of a protein or structural or other genetic mutations.

**Fig. 5:**
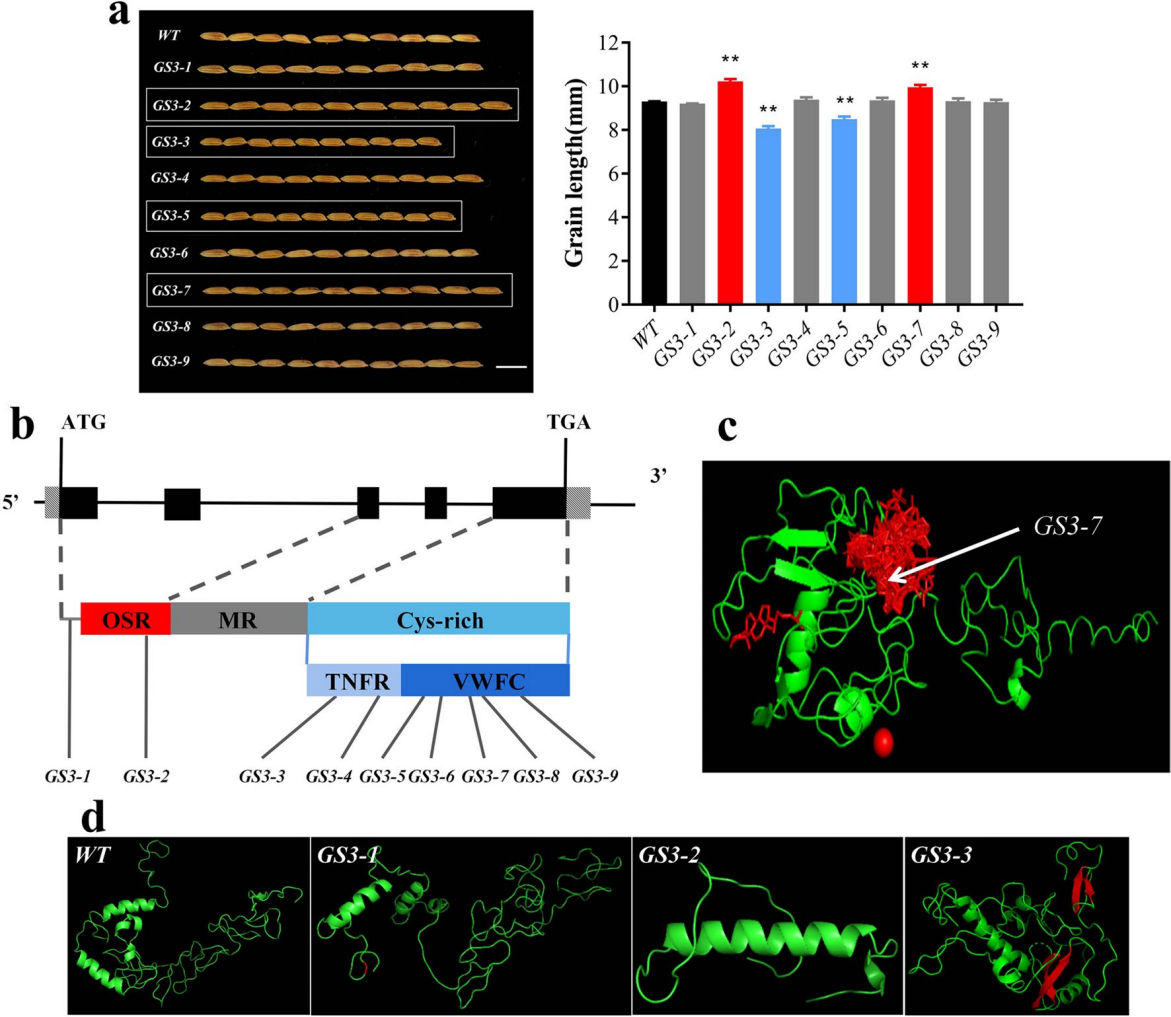
**a** Grain length and WT alignment of nine *GS3* mutants. Scale bars = 1 cm. **b** Mutant distribution on gene and protein structure. **c** *GS3* protein functional site (red part). **d** Protein structures of *GS3* mutants (differential structures in red)

We screened a total of 9 grain length mutants, of which 2 grain lengths were significantly longer and 2 grain lengths were significantly shorter than WT grains (Fig. 5a). Nine SNPs related to *GS3* were identified, including 8 nonsynonymous mutations and 1 nonsense mutation (*GS3-2*), in which *GS3-1* was located in exon 1 and *GS3-2* was located in exon 2. The remaining seven mutations were located in exon 5 (Fig. 5b). The mutation position of *GS3-1* is relatively advanced, and it is not located in the functional structural region and has no effect on the structure and function of the protein, so the grain length does not change significantly (Fig. 5b). *GS3-2* is located in the OSR domain, the 55th amino acid is mutated to a stop codon, the OSR domain is deleted, and the protein structure and function are severely affected (Fig. 5d), resulting in a significant increase in grain length. Both *GS3-3* and *GS3-4* are located in the TNFR domain of Cys-rich mutants, and the grain length of *GS3-3* is significantly reduced. Protein structural analysis showed that the mutation of amino acid No. 135 leads to two additional β sheets in the secondary structure of the protein. and presumably resulted in impaired TNFR domain function (Fig. 5d), whereas the *GS3-4* grain length was not significantly altered. *GS3-5*, *GS3-6*, *GS3-7*, *GS3-8* and *GS3-9* are all located in the Cys-rich VWFC domain, among which only the grain length of *GS3-5* is significantly reduced, and the mutation of *GS3-5* may lead to the impaired structure of the VWFC domain function, but there is no significant difference in protein structure compared with *WT* plants (Additional file 3: Fig. S3). The *GS3-6*, *GS3-8* and *GS3-9* phenotypic results were similar to those of *GS3-4*; functional domain amino acid point mutation occurred, but the phenotype did not change significantly. It was speculated that the mutation of these 4 amino acids may not affect the function of the protein or that other gene mutations have an impact on the phenotype; however, the grain length of *GS3-7.* In contrast, the functional site analysis of its protein showed that the mutation of amino acid 183 of *GS3-7* was located in the ligand binding site of the protein (Fig. 5c). After the mutation, the function of the protein was affected, so the particle shape changed. However, *GS3-4*, *GS3-5*, *GS3-6*, *GS3-8* and *GS3-9* showed no significant difference when compared to the *WT* in terms of protein structure and function (Additional file 3: Fig. S3).

**Table 3:** Amino acid mutation and phenotypic information of single mutant plants

Mutant number	Single plant number	Amino acid mutation	Grain length (mm)	Grain width (mm)
*WT*	WT		9.31	2.03
*GS3-1*	7-112-2-4	A5G	9.21	2.12
*GS3-2*	4-101-1-3	C55X	10.22**	2.03
*GS3-3*	5-6-1-4	C135W	8.06**	2.04
*GS3-4*	4-101-2-2	S141C	9.41	2.17
*GS3-5*	6-23-1-4	C173F	8.51**	1.9
*GS3-6*	7-78-1-3	G167C	9.33	1.94
*GS3-7*	9-5-1-1	C190F	9.97**	1.86
*GS3-8*	7-78-2-3	S184F	9.33	2.09
*GS3-9*	7-41-1-4	R200H	9.30	1.9
*GW5-1*	5-35-1-2	V411G	9.53	2.29**
*GW5-2*	7-82-1-4	A405G	9.42	2.02
*GW5-3*	10-14-2-1	K397E	9.46	2.03
*GW5-4*	10-51-2-3	D97G	9.40	1.84**

*: represents significant variation at *p* < 0.05; **: represents highly significant variation at *p* < 0.01

**Fig. 6:**
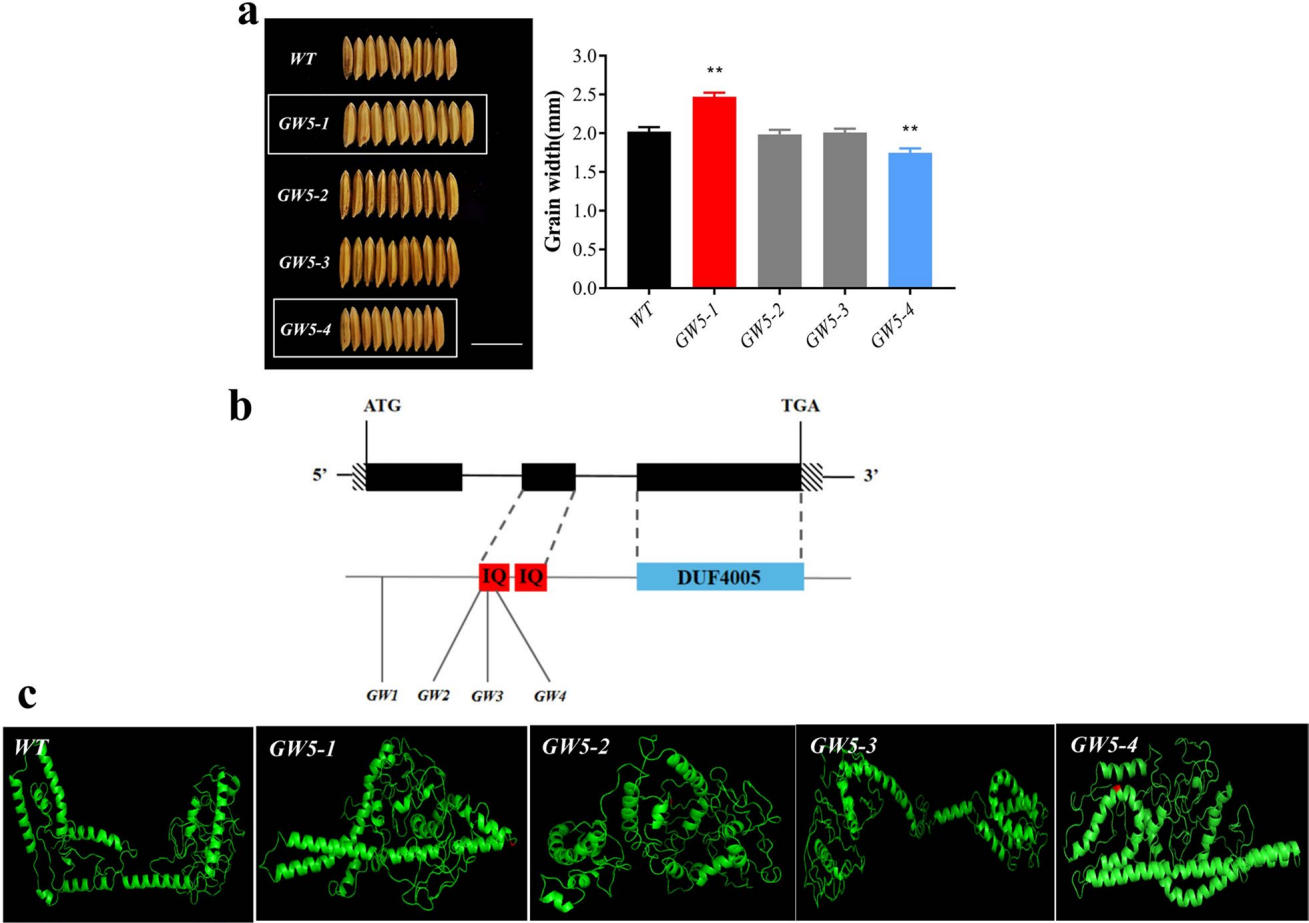
**a** Grain width and *WT* alignment of four *GW5* mutants. Scale bars = 1 cm. **b** Mutant distribution on gene and protein structure. **c** Protein structures of *GW5* mutants (differential structures in red)

We screened 4 mutants with grain width, of which 1 grain width increased, 1 grain width decreased, and the remaining two grain widths had no significant changes (Fig. 6a). The four identified *GW5*-related SNPs were all nonsynonymous mutations, of which *GW5-4* was located in exon 1, and the other three were located in exon 2 (Fig. 6b). *GW5-1*, *GW5-2* and *GW5-3* are all located in the calmodulin-binding domain; the difference is that only the granule width of *GW5-1* is significantly wider than that of other mutants, and the other two mutants have no obvious change in phenotype. We predicted the protein structure and function of mutants and found that the protein structures of *GW5-1* and *GW5-2* were more similar to each other than to the *WT*, while the protein structure of *GW5-3* had no obvious change (Fig. 6c). The phenotypes of *GW5-1* and *GW5-2* of the same domain differ, presumably due to interference from other genes. The *GW5-4* mutation position is relatively forward, not located in the functional structural region, and has no effect on the structure and function of the protein (Fig. 6b), but the grain width is significantly narrowed, and the protein structure is relatively concentrated (Fig. 6c).

### Whole Genome Sequencing of Key Mutants Identifies New SNPs Affecting the ***GS3*Mutation Effect**.

**Fig. 7:**
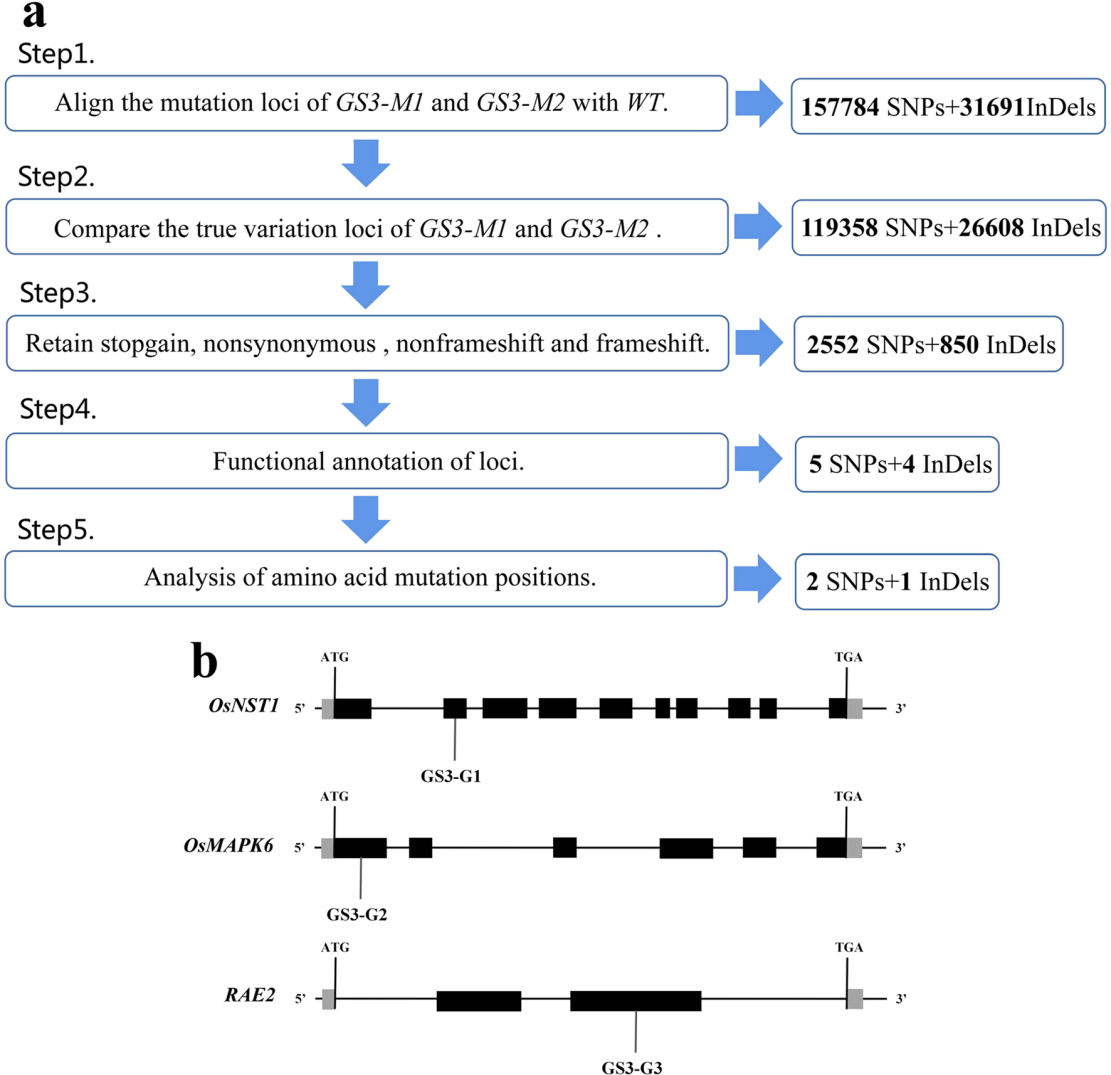
**a** Whole-genome mutation screening process. **b** Positions of the three new SNPs on their corresponding genes

To explore the reasons for the contradiction between genotype mutation and phenotype mutation and to clarify whether allelic variation in other grain length-related genes had an impact on the phenotype, GS3-M_1_, GS3-M_2_ and WT sample were subjected to WGS. A total of 2,084,534 SNPs and 336,039 InDels were obtained by sequencing GS3-M_1_, and 2,116,343 SNPs and 341,777 InDels were obtained from GS3-M_2_. There were 189473 different loci in the two samples after comparison (Additional file 10; Table S7). After screening, three new allelic variants related to grain length were finally obtained (Table 4) (Fig. 7a). GS3-G1 is located in the second exon of *OsNST1*, which mutates serine No. 65 to threonine. At present, there are few reports on this gene, and its protein structure cannot be predicted. Mutants exhibit reduced cell wall cellulose content and structural changes, resulting in reduced mechanical strength and abnormal plant development, such as dwarf plants and smaller seed size (Song et al. 2011). GS3-G2 is a variant located in the first exon of *OsMAPK6* that mutates the aspartic acid at No. 131 to arginine, which affects only one of its functional domains. Inhibition of *OsMPK6* expression can make rice panicles denser and grains smaller, and mutation of this gene can significantly reduce grain length, grain width and thousand-grain weight (Guo et al. 2018). GS3-G3 is located in the second exon of *RAE2*, resulting in a frameshift insertion mutation at amino acid 99 and impaired function of the cysteine-rich region of the encoded protein EPFL1. The number of kernels decreased, the kernels became longer, and the proportion of awned kernels increased (Jin et al. 2016) (Fig. 7b).

**Table 4:** Whole-genome mutation site information

Number	Chr	Location	Ref	Alt	Structure gene	Function type
GS3-G1	2	24,236,917	C	T	Os02g0614100	Nonsynonymous SNV
GS3-G2	6	2,812,755	T	C	Os06g0154500	Nonsynonymous SNV
GS3-G3	8	23,999,540	–	C	Os08g0485500	Frameshift insertion

## Discussion

### Reliability of MTWA to Identify Mutants and Allelic Variants

Screening mutants and identifying allelic variants are important foundations for innovative germplasm materials and functional genomics research (Guo et al. 2006). To efficiently utilize germplasm resources, the most fundamental way is to excavate new alleles and purposefully aggregate or transfer them in conventional breeding or molecular breeding and then combine them with molecular design to achieve the purpose of improving breeding efficiency. In this study, the seeds of Huahang 31 were irradiated with ^12^C^6+^, and the M_2_ generation population containing 3872 individual plants was obtained. The variation coefficient of grain length was 1.89%, and the variation coefficient of grain width was 0.56%, indicating that there are many potential mutations in grain type. body. In this study, a new method for allelic variation and mutant identification, MTWA, was proposed, which used targeted sequencing technology to initially identify mixed sample materials. After screening, a total of 15 SNPs and 12 mixed sample materials were obtained, and Sanger sequencing was performed. The key mutants in the mixed samples were screened out and verified for authenticity. The consensus rate between targeted sequencing and Sanger sequencing was 86.67%, proving the feasibility of the method. A total of 13 mutants and 15 SNPs were screened out. Analysis of the phenotype of the mutant individual plant, the protein functional structural analysis of the mutation site, and WGS of the key mutants were conducted. Mining new grain length-related allelic variations, analyzing the connection between genotype mutations and phenotype mutations, and establishing a set of systematic, efficient and accurate new methods for allelic variation identification were also conducted. At the same time, a batch of mutation sites and mutant materials with breeding value were screened and identified.

### Advantages of MTWA

Traditional forward genetic identification methods usually require the construction of complex mapping populations and linkage maps and gene linkage analysis using genetic markers (Zhang et al. 2012; Serquen et al. 1997; Fazio et al. 2003) but can only map mutation sites to a large range of chromosomal regions (Hazen 2005), and fine mapping of mutant genes is expensive and time consuming (Schneeberger et al. 2009; Abe et al. 2012). MTWA can be utilized to analyze the M_2_ generation without the need to construct a complex genetic population, which greatly shortens the detection time. This technique is also a directional mutant identification method. TILLING technology uses CEL I enzyme to digest PCR amplification products and detects and selects mutants by capillary electrophoresis (Yan et al. 2014), but the process is relatively complicated; however, the TILLING technique can detect gene fragments. There are certain length requirements; usually, the length of the target gene fragment is less than 1.5 kb, and the high-resolution melting curve (HRM) detection region is only 150–500 bp (Sikora et al. 2011). On the other hand, MTWA directly performs second-generation sequencing on the target fragment to determine the mutation sites. The MTWA process is relatively simple, and the sequencing results are more accurate than those of the TILLING method. At the same time, multiple mutation sites can be detected in a single amplicon, and there is no restriction on the fragment length. The detection efficiency is higher and has wider applicability than the TILLING method. Compared to MutMap and its derived methods, mutants in MTWA do not require backcrossing of parental lines, thus greatly reducing time and effort (Allen et al. 2013; Abe et al. 2012); if the genome of the target crop is large and complex, MutMap will have the problems of high sequencing costs, large datasets, and difficult comparison and analysis, especially in allopolyploid species with high genome heterozygosity, and highly homologous sequences and subtypes of genomes could also be detected (Li et al. 2015; Ling et al. 2013; Michael et al. 2018; Consortium 2014). As a mutant and allelic variant directional identification method, MTWA only needs to sequence the target fragments of mixed samples in the early stage, and the number of WGS samples of key mutants in the later stage is small, so the costs of sequencing and the data analysis are greatly saved. These mutants can be detected in the M_2_ band, and the detection duration of this method is short and not limited to the particle type phenotype, allowing for capturing and detecting any target fragment with high flexibility.

### The Breeding Significance of Mining New Allelic Variations

A total of 13 grain type-related SNPs were identified in this study, of which 6 SNPs had an impact on phenotypic changes, which can be used to develop molecular markers; 7 SNPs that did not affect phenotypes can also provide theories for follow-up research via nonsense allelic variation studies. At the same time, we screened a batch of germplasm materials with obvious differences in grain shape from the mutagenized progeny, such as the long-grain mutants *GS3-2* and *GS3-7*, short-grain mutants *GS3-3* and *GS3-5*, wide-grain mutant somatic *GW5-1* and narrow-grain mutant *GW5-4*. Among these mutants, the genotype mutation of *GS3-2* contributed 9.77% to the grain length, which was consistent with the mutation position of the mutant obtained by Mao et al. (2010), indicating that the allelic mutation was reliable and the mutation mechanism was clear; *GW5-4* mutation led to a 13.43% reduction in grain width and was the site with the largest variation in grain shape among all mutations. These two sites can be used as key SNPs to develop molecular markers and provide new functional genes for molecular breeding. locus, serving breeding practice.

*GS3* encodes a transmembrane protein consisting of 232 amino acids. The protein product consists of three conserved domains: OSR, MR and Cys-rich domains. The Cys-rich domain includes two regions, TNFR/NGFR and VWFC. The OSR domain plays an important role as a negative regulator, and loss of the OSR structure and function results in the formation of long grains. In *GS3-2* identified in this study, the 55th amino acid of the OSR domain was mutated to a stop codon, and the OSR domain was deleted. The protein structure and function were severely affected, resulting in a significant increase in grain length. This mutation is also similar to that of previous studies. The results of the study of *GS3-5* are the same as those of previous studies, moreover, the mutation of *GS3-5* may lead to the impaired function of the VWFC domain, which is also consistent with previous conclusions, but the protein structure is not obvious, *GS3-7* also resulted in impaired VWFC domain function but increased grain length, contrary to previous conclusions (Mao et al. 2010). Additionally, in the mutation of *GW5*, *GW5-1* and *GW5-2* are in the same domain, but the phenotypes are different, while *GW5-4*, which is not located in the functional domain, has a significantly reduced grain width. Therefore, we speculate that the mutants with phenotypic variation identified in this study that do not match the genotypic variation may have other grain-related variants that have an impact on the phenotype.

### Analysis of the Effect of New Grain Length-Related SNPs on the Effect of *GS3* Mutation

In this study, using targeted sequencing, Sanger sequencing combined with protein analysis, we have determined the association between some loci and granulotype variation, such as *GS3-1*, *GS3-2*, *GS3-3 GW5-1* and *GW5-4* etc. However, there are some contradictions between loci and phenotypic traits, such as *GS3-4*, *GS3-5*, and *GS3-7*. To explore whether there are new grain-length gene variants for expression, we found new allelic mutations in GS3-G1, GS3-G2 and GS3-G3 by WGS. The protein structure of *GS3-5* was not significantly different from that of WT, but the grain length was significantly reduced, it is speculated to be due to the effect of the mutation of GS3-G1 or GS3-G2 locus. The GS3-G1 mutation will lead to a significant reduction in grain size, and the 1000-grain weight is reduced to 50% of WT (Zhang et al. 2011). GS3-G2 site will result in a 20% reduction in grain length (Guo et al. 2018), so the presence of GS3-G1 or GS3-G2 sites ultimately results in a reduction in grain length in *GS3-5*; *GS3-7* The mutation type of GS3-G3 will theoretically lead to a decrease in grain length, but the actual grain length increases by 7.09%, while the mutation at the GS3-G3 site will lead to an increase of about 8% in grain length (Jin et al. 2016). The phenotypic mutation effect is greater than that of *GS3-7*, so the final response is the grain length in the phenotype. An increase in *GS3-4*, *GS3-6*, *GS3-8*, and *GS3-9* genotype mutations will theoretically lead to reduced grain length, but the actual grain length of these mutants was not significantly different from that of the WT plants, presumably due to the mutation of GS3-G3. The increase in grain length counteracted the phenotypic mutation effect of the original *GS3* mutant, so the result reflected in grain length was no significant change in phenotype. Grain type is a relatively complex trait. Although *GS3* and *GW5* have a great influence on grain shape, there may also be other genes that affect grain shape changes at the same time. Therefore, we used WGS to propose this possibility, and analyzed the new genes and SNPs on phenotype are described, making the MTWA method more complete and reliable.

## Materials and methods

### Material Irradiation, Planting and Collection

The WT material used in this experiment was Huahang 31, and the dry seeds of rice were irradiated with the high-energy ion beam ^12^C^6+^ provided by the HIRFL of the China Institute of Modern Physics, Lanzhou. The irradiation energy was 80.55 MeV/u, the dose was 80 Gy, and the dose rate was 60 Gy/min. Simultaneously, unirradiated seeds were prepared as controls, and a total of 100 g of seeds (about 5000 seeds) were irradiated. The irradiated M_0_ generation seeds and the control were planted in the South China Agricultural University base in July 2017, and the single main ear of the M_0_ generation was harvested. The mutagenic generation after mixing was M_1_, and the M_1_ generation was continued to be planted to harvest the main ear and mixed to obtain M_2_. The seeds of the M_2_ generation were harvested for the main ear (about 200 seeds) per plant, and the corresponding leaves per plant were harvested at the same time. The seeds and leaves were sun-dried and stored in a −20 °C refrigerator.

### Grain Type Phenotypic Measurements and Data Analysis

30 seeds were taken from each M_2_ generation per plant, and the grain shape images were taken with a scanner, and then the images were analyzed with SmartGrain (software version. 1.2) to obtain the phenotypic data of grain length and grain width. The obtained granulotype data were statistically analyzed with SPSS (Statistical Analysis System, version 23.0), and the mean, standard deviation and coefficient of variation were calculated, and then GraphPad prism, version 7 for Windows (GraphPad Software, CA, USA) was used to draw the frequency distribution diagram of granulotype data.

### Extraction and Quantification of DNA

Take about 100 mg of each M_2_ single leaf, and DNA was extracted by the CTAB (Murray and Thompson 1980). The concentrations of DNA were determined using Qubit and NanoDrop (Thermo Fisher Scientific, Wilmington, DE, USA). DNA samples that passed the quality inspection were stored at −80 °C for subsequent experiments.

### Targeted Sequencing

A total of 484 mixed samples and 1 WT sample were obtained by mixing 3872 individual DNAs in equal amounts at a ratio of 8:1. The detected fragments were the main gene *GS3* (Fig. 3a) of rice grain length and the main gene of grain width *GW5* (Fig. 3b). Firstly, specific amplification primers were designed for multiple SNP loci to be tested (Additional file 4: Table S1), nonspecific amplification were suppressed in the first round of PCR, and targeted primers were enabled to achieve highly uniform amplification in one tube of PCR, thereby enriching the target fragments in large quantities. Subsequently, in the second round of PCR, sequencing adapters and library barcodes were added to finally obtain the library required for sequencing. Finally, the marker genotypes of the target loci were revealed by massively parallel sequencing (Fig. 3c).

### Screening for Mutation Sites

After the targeted sequencing was completed, the VAF of the SNP site was first analyzed and calculated (the calculation method is shown in Additional file 1: Fig. S1), and the WT sample was used as a reference to screen for mutation sites: (1) When the parent is a pure genotype at this site, the offspring and the parent are at this site; (2) when the parent is a heterozygous genotype at this locus and the mutation frequency difference between the offspring and the parent at this locus is greater than or equal to 1/16, the locus shall be retained.

### Sanger Sequencing

We added 200 bp before and after each of the 15 SNPs and used Primer3 (https://bioinfo.ut.ee/primer3-0.4.0/) design 15 pairs of primers (Additional file 5: Table S2), and performed PCR amplification on the individual material corresponding to each SNP to obtain Sanger sequencing was performed after the target fragment, and the sequencing data was compared with the wild type by SnapGene (https://www.snapgene.com/), and the peak map was drawn.

### Identification of Mutants Authenticity

In order to verify the authenticity of the selected mutants, we identified the authenticity of the 13 single-plant materials screened according to the Protocol for identification of rice varieties-SSR marker method (NY/T 1433–2014) issued by the Ministry of Agriculture and Rural Affairs of People’s Republic of China, and selected 10 pairs of SSR markers and designed the corresponding primers using Primer3 (https://bioinfo.ut.ee/primer3-0.4.0/), then the PCR productions were detected by 1% agarose gel electrophoresis (Additional file 6: Table S3).

### Protein Structure and Function Analysis

For the amino acid mutations corresponding to the identified SNPs, the functional site of the protein was predicted by I-TASSER (https://zhanggroup.org/I-TASSER/), and then the functional site of the protein was predicted by Phyre2 (http://www.sbg.bio.ic.ac.uk/phyre2/html/) to predict the three-dimensional structural model of proteins. PyMOL version 2.4.1 (New York, NY, USA) was used to draw protein functional sites and three-dimensional structure maps and perform annotation and comparison.

### Whole Genome Sequencing

We used the mutant *GS3-5* and *GS3-7*, DNA was extracted, and equal amounts of DNA were mixed to construct mixed sample GS3-M_1_; mutants *GS3-4*, *GS3-6*, *GS3-8*, and *GS3-9* with genotype mutation but no change in phenotype were utilized to construct mixed sample GS3-M_2_; and *WT* plants were utilized to prepared the WT sample. A total of 3 samples were subjected to Whole Genome Sequencing. The genomic DNA was randomly broken into short DNA fragments with enzymes and then blunt-end repaired. dA tails were ligated to both ends of the DNA fragments, and sequencing adapters were ligated. The DNA fragments with adapters were purified by AMPure XP (Beckman Coulter, CA, USA) magnetic beads, and fragments in the range of 300–400 bp were selected for PCR amplification. The constructed library was purified, checked against a library, and sequenced on a the Illumina HiSeq 2500 Sequencing Systems Platform (Illumina Inc. USA).

### Sequencing Data Filtering and New SNP Acquisition

Fastp (Chen et al. 2018) software was used to filter the raw reads to remove the adapter sequence (adapter); when the N content contained in the sequencing read exceeded 10% of the length of the read, the paired reads needed to be removed. When the number of bases of quality (Q ≤ 20) exceeded 40% of the length of the read, the paired reads needed to be removed. Variation detection was performed using GATK (McKenna et al. 2010) software. When the coverage depth of a sample at a certain SNP site was less than 5×, the sequencing depth of the sample at this site was insufficient. To ensure the accuracy of genotyping results, the site is treated as a deletion. In the parental sample, if VAF (variant allele frequency) ≥ 0.8 or ≤ 0.2, the locus was a pure-sum mutation, and if the mutation frequency was between 0.2 and 0.8, the SNP locus was a heterozygous mutation. The mutation sites of GS3-M_1_ and GS3-M_2_ were compared with WT, the WT sites were removed, and the true variation was retained; the true variation in GS3-M_1_ and GS3-M_2_ was compared with each other to determine the difference sites between the two. Remove heterozygous sites, retain homozygous sites, while retaining nonsynonymous, nonsense, frameshift, and non-frameshift mutations that cause phenotypic variation at differential sites, only nonsynonymous mutations, nonsense mutations, frameshift mutations and nonframeshift mutations that cause phenotypic variation in the differential sites were retained. Functional annotation was performed on the points, focusing on the sites related to grain length. The amino acid mutation positions of the selected sites were analyzed, and only the sites that caused the functional changes in the protein domain were retained.

## Supplementary Information

**Additional file 1: Fig. S1.** VAF calculation method, green bases are mutation bases, red bases are reference genome bases, and the ratio of green bases to all bases is the VAF.

**Additional file 2: Fig. S2.** The Sanger sequencing results of the mutation sites of the 15 mutant individual plants, in which the results of SNP-5 and SNP-6 are different from the targeted sequencing results, these two mutant individual plants are excluded, and the rest are the same as the targeted sequencing results, which are true variation.

**Additional file 3: Fig. S3.** Protein structures of 9 *GS3* mutants, the red part is the difference from *WT.*

**Additional file 4: Table S1.** Targeted sequencing primer information.

**Additional file 5: Table S2.** Primers for amplifying fragments of the target site.

**Additional file 6: Table S3.** Primers for authenticity verification of mutant individuals.

**Additional file 7: Table S4.** Grain type datas.

**Additional file 8: Table S5.** Mutation mixed sample information screened by targeted sequencing.

**Additional file 9: Table S6.** Mutation site information obtained by targeted sequencing.

**Additional file 10: Table S7.** Mutation site information screened by whole genome sequencing.

## Data Availability

All data generated or analyzed during this study are included in this published article and its supplementary information files.
