# Supplementary figures and images for: Targeted Identification of Rice Grain-Associated Gene Allelic Variation Through Mutation Induction, Targeted Sequencing, and Whole Genome Sequencing Combined with a Mixed-Samples Strategy
Source: Rice (N Y). 2022 Nov 3;15:57. doi: 10.1186/s12284-022-00603-2 (PMC9633910; doi:10.1186/s12284-022-00603-2)

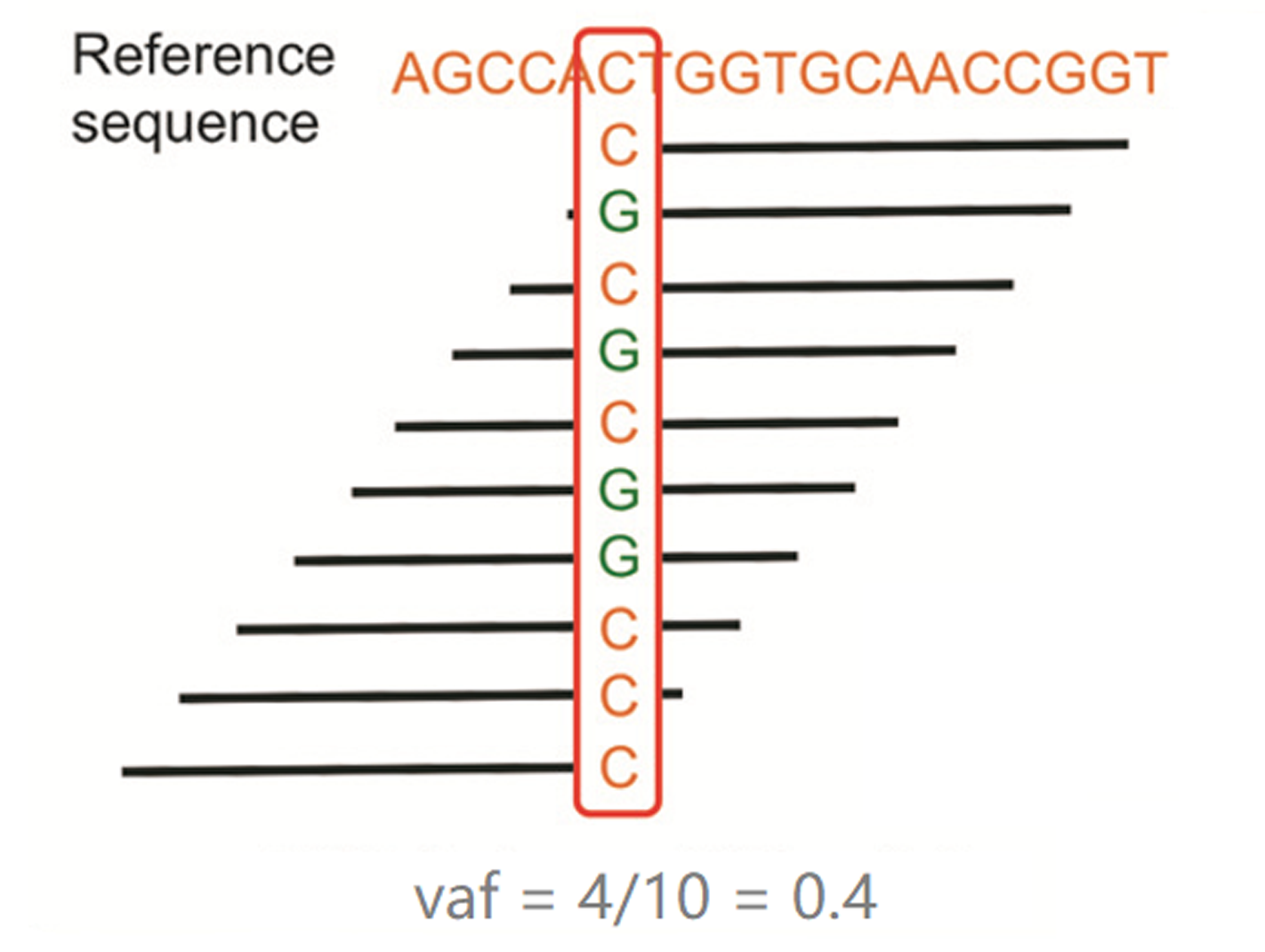

Supplement: Supplementary file 1 — Additional file 1: Fig. S1. VAF calculation method, green bases are mutation bases, red bases are reference genome bases, and the ratio of green bases to all bases is the VAF. [file 12284_2022_603_MOESM1_ESM.tif]

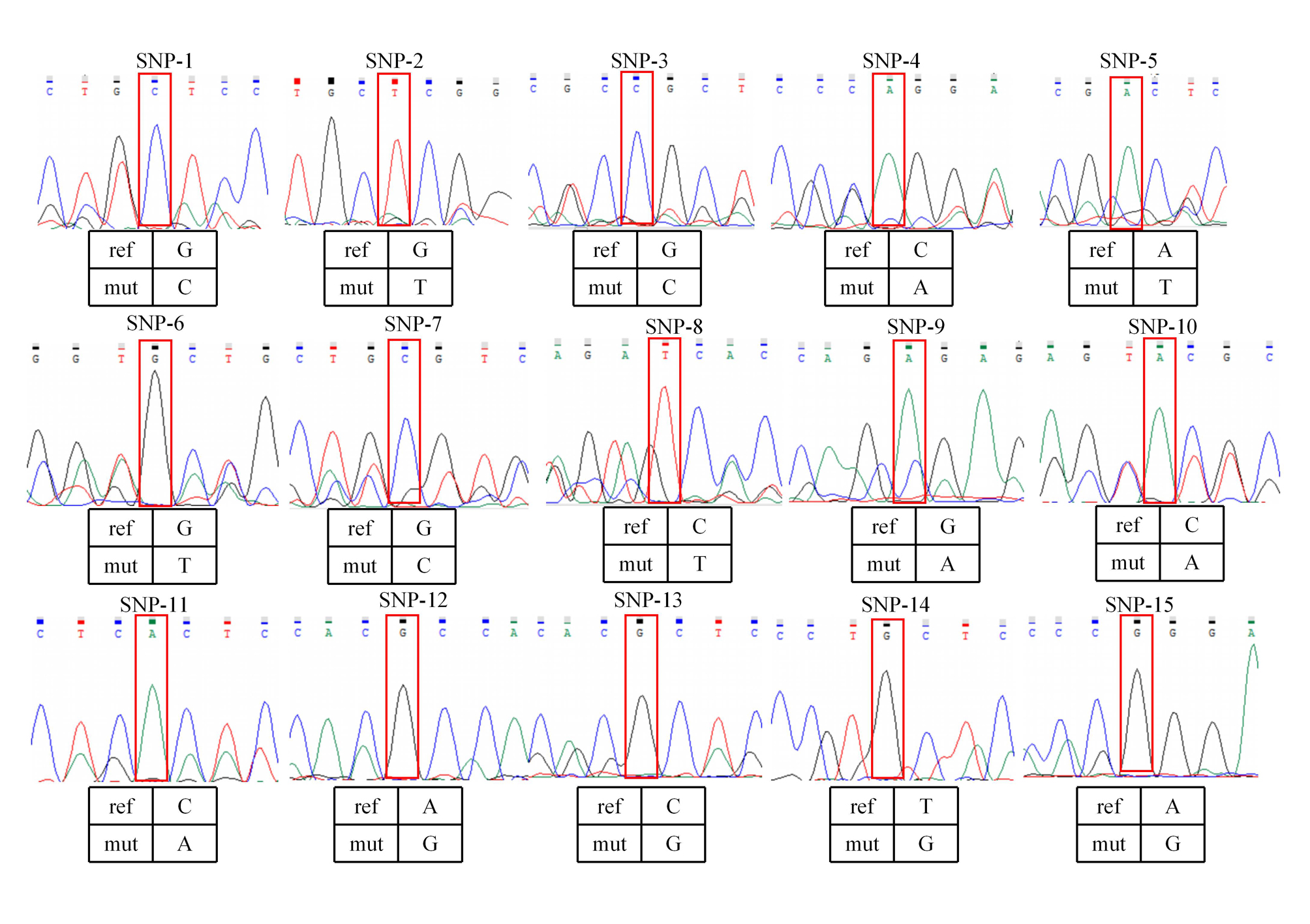

Supplement: Supplementary file 2 — Additional file 2: Fig. S2. The Sanger sequencing results of the mutation sites of the 15 mutant individual plants, in which the results of SNP-5 and SNP-6 are different from the targeted sequencing results, these two mutant individual plants are excluded, and the rest are the same as the targeted sequencing results, which are true variation. [file 12284_2022_603_MOESM2_ESM.tif]

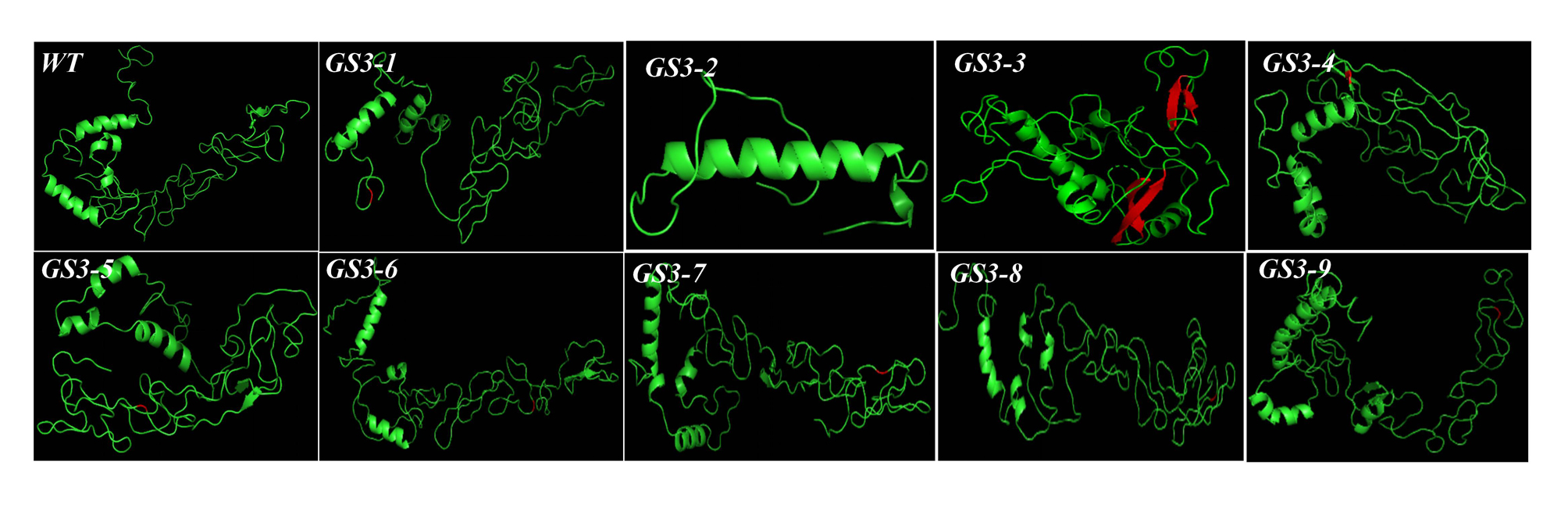

Supplement: Supplementary file 3 — Additional file 3: Fig. S3. Protein structures of 9 GS3 mutants, the red part is the difference from WT. [file 12284_2022_603_MOESM3_ESM.tif]
